# High sensitivity C-reactive protein in pre-eclamptic women living with HIV at a tertiary hospital in Zambia: a preliminary study

**DOI:** 10.11604/pamj.2024.48.136.42683

**Published:** 2024-07-25

**Authors:** Zebedia Kabaya, Rehana Omar, Andrew Kumwenda, Musalwa Muyangwa-Semenova, Moses Mukosha

**Affiliations:** 1 Department of Physiological Sciences, School of Medicine, University of Zambia, Lusaka, Zambia,; 2 Department of Obstetrics and Gynaecology, School of Medicine, University of Zambia, Lusaka, Zambia,; 3 School of Public Health, Faculty of Health Sciences, University of Witwatersrand, Johannesburg, South Africa,; 4 Department of Pharmacy, School of Health Sciences, University of Zambia, Lusaka, Zambia

**Keywords:** High sensitivity C-reactive protein, pre-eclampsia, HIV-infection, Zambia

## Abstract

**Introduction:**

pre-eclampsia affects an estimated 8% of pregnant women and contributes to over 12% of global maternal deaths. High-sensitivity C-reactive protein (hs-CRP) is a potential marker of pre-eclampsia. However, little is known about hs-CRP levels in women with pre-eclampsia in Zambia. This study examined whether hs-CRP levels differ between women who develop pre-eclampsia compared with controls overall and in subgroups of women living with and without HIV.

**Methods:**

a case-control study was conducted among 40 pregnant women who developed preeclampsia (cases) and 40 normotensive pregnant women (controls) living with (n=20) and without HIV (n=20) at women and newborn hospital from February to May 2022. Standard ELISA kits were used to determine hs-CRP levels. The conditional logistic regression model calculated the odds ratios for hs-CRP and other predictor variables with their 95% confidence intervals.

**Results:**

the median hs-CRP levels were higher among the cases than controls (7.84mg/ml vs 6.13mg/ml, p<0.001). Similar hs-CRP levels were observed among pre-eclamptic women living with HIV on antiretroviral therapy (ART) compared to HIV-negative women (7.92mg/ml vs 7.17mg/ml, p=0.862). On the other hand, normotensive women living with HIV on ART had different hs-CRP levels than HIV-negative women (6.60mg/ml vs 3.96mg/ml, p<0.001). Multivariable conditional logistic regression showed that pregnant women with higher levels of hs-CRP (AOR=1.01, 95% CI=1.01, 1.01) were more likely to have pre-eclampsia after adjusting for significant predictors. Pre-eclampsia was less likely among women living with HIV on ART (AOR=0.26, 95% CI=0.07, 0.99), married (AOR=0.15, 95% CI=0.03, 0.71), and multiparous (AOR=0.16, 95% CI=0.03, 0.80).

**Conclusion:**

high-sensitivity C-reactive protein levels were higher among the cases than controls. However, similar levels were observed in the subgroup of women living with HIV on ART. Participants with high hs-CRP levels had the highest odds of preeclampsia, suggesting that hs-CRP may be useful in predicting preeclampsia.

## Introduction

Pre-eclampsia, a pregnancy-specific disorder diagnosed after 20 weeks of gestation, affects 8-10% of pregnant women globally [1]. Low and middle-income countries (LMICs) share this burden disproportionately, with over half of the cases occurring in this region [2]. In Zambia, pre-eclampsia occurs in 12% of pregnant women [3]. Pre-eclampsia is one of the main causes of morbidity and mortality among pregnant women [4]. Additionally, women with human immunodeficiency virus (HIV) infection receiving antiretroviral therapy (ART) are thought to be at a higher risk of pre-eclampsia compared to their HIV-negative counterparts [5-7]. One study in Zambia showed a 1.27-fold increase in the odds of developing hypertensive disorders of pregnancy among HIV-infected women receiving ART compared to HIV-negative women [8].

Pre-eclampsia is a multisystem vascular disorder of pregnancy classified as either mild or severe based on the degree of symptoms resulting from the involvement of systemic organs [9]. According to the American College of Obstetricians and Gynaecologists (ACOG) [10], pre-eclampsia is defined as the presence of blood pressure greater than or equal to 140/90 mmHg after 20 weeks gestation with proteinuria of ≥ 300 mg/24hr or ≥ 1+ dipstick or new onset of other end organ damage such as the liver or kidneys. The causes of pre-eclampsia remain unknown, but many factors such as multiple gestations, diabetes, and first pregnancy, are associated with its development [11]. Recent studies have linked pre-eclampsia to HIV infection and ART, with conflicting results. Some have reported a lower risk, whereas others have reported a higher risk or no difference [3,12-15].

While several hypotheses have been generated to explain the association between HIV infection and pre-eclampsia, understanding of the underlying mechanisms remains limited [16]. Both pre-eclampsia and HIV infection are inflammatory conditions. At the time of diagnosis, women with pre-eclampsia and HIV infection have profound disruption of the endothelium and increased activation of systemic inflammation [17,18]. This disruption is manifested in the abnormal expression of markers of inflammation, such as high-sensitivity C-reactive protein (hs-CRP) [11,19]. High-sensitivity C-reactive protein is a 206 amino acid protein member of the pentraxin family of acute phase proteins that increase markedly in infectious diseases and inflammation [20,21]. It is secreted and synthesized by hepatocytes in the liver in response to cytokines, interleukin-6 (IL-6), and tumour necrosis factor-alpha (TNF-α) [22].

In the general population, studies have reported a significant increase in hs-CRP levels in the first trimester in pregnant women with pre-eclampsia than in normotensive counterparts [23,24]. Few studies have assessed the association between the levels of hs-CRP and pre-eclampsia in high HIV prevalence settings. To address this gap, this study examined whether hs-CRP levels differ between women who develop pre-eclampsia compared with controls overall and in subgroups of women living with and without HIV.

## Methods

**Study design, setting, and population:** the case-control study was conducted between February 2022 and May 2022 at the Women and Newborn Hospital in Lusaka, Zambia. The Women and Newborn Hospital is the biggest national referral hospital and leading institution in maternal and reproductive health in Zambia with a bed capacity of 453 and attending to diverse pregnancy complications [25]. At the study setting pre-eclampsia is diagnosed according to the ACOG criteria as a new-onset of hypertension (systolic blood pressure ≥140 mm Hg and diastolic blood pressure ≥90 mm Hg) after 20 weeks of gestation and the presence of proteinuria or with other maternal organ dysfunction with no evidence of urinary tract infection (UTI) in a random urine sample [10]. This study included 80 pregnant women. Pregnant women diagnosed with pre-eclampsia and normotensive women. The study further stratified the participants into subgroups based on HIV serostatus (pre-eclamptic HIV-negative, pre-eclamptic HIV-positive, normotensive HIV-negative, and normotensive HIV-positive). Pregnant women with conditions such as diabetes, cardiac disease, chronic hypertension, epilepsy, chronic renal disease, sickle cell disease, abruptio placentae, antiphospholipid antibody syndrome, chorioamnionitis, eclampsia, thyroid disorder which would affect the levels of hs-CRP, and unknown HIV status were excluded.

**Enrollment procedure:** two nurse research assistants were trained on the study procedures to help with enrolment and blood sample collection. The research assistants contacted the pregnant women who met the inclusion criteria from the antenatal care ward and high dependent unit (HDU) only after a thorough medical examination by the obstetrician on duty. The purpose of the study, its potential benefits, and any risks involved were explained to the participants by the researcher and research assistants. Pregnant women ≥ 20 weeks gestation admitted to the women and newborn hospital were eligible. During the study, at least 5-10 pre-eclamptic patients were seen daily. Study staff used a systematic sampling technique to enroll cases and controls. On each day of enrolment, a pregnant woman was selected randomly, and then every 4^th^ person who met the inclusion criteria was selected until the desired sample size was achieved. The outcome variable was pre-eclampsia measured on a binary scale (yes=1, no=0). A case was defined as a pregnant woman diagnosed with pre-eclampsia. Pre-eclampsia was diagnosed if a participant presented with systolic blood pressure above 140 mm Hg or diastolic blood pressure above 90 mmHg with proteinuria or the presence of any end organ damage. Proteinuria of 1 or 2 + on the urine dipstick was taken as positive. A control was defined as a pregnant woman without hypertension or proteinuria but attending regular antenatal wards. The cases and controls were matched on age group (between 20 to 41 years).

**Study exposures:** the primary exposure was the high-sensitivity C-reactive protein levels measured using blood samples (measured on a continuous scale). HIV serostatus (positive/negative) was determined through medical records. In addition, the study measured sociodemographic, clinical, and obstetric characteristics, including age (years), marital status (married/unmarried), parity (multiparous/nulliparous), gestational age (20-30 years/above 30 years), urea (mmol/l) and creatinine (µmol/l). In this study, pre-pregnancy body mass index (BMI) was calculated based on the weight of the reported weight before pregnancy and was categorized according to WHO criteria as normal (BMI < 25 kg/m^2^), overweight (25 < BMI < 30 kg/m^2^) and obese (BMI ≥ 30 kg/m^2^) [26].

**Determination of high-sensitive human C-reactive protein (CRP) using ELISA:** venous blood was collected during antenatal visitation and rounds in the labour ward and high-dependent unit wards. Five milliliters of blood was drawn from the vein on the antecubital area of the arm using a sterile needle and syringe into a purple ethylene diamine tetra acetic acid (EDTA) anti-coagulant vacutainer blood collection tube. The vacuum tubes were labeled using the patient's unique participant ID number and placed in a cold chain (icebox). The blood was transported within 30 minutes to the laboratory. The blood was then centrifuged at 3000 rpm for 3 minutes, and the resulting serum was drawn and stored in serum tubes (plain containers) at -80°C at the clinical chemistry laboratory of the University Teaching Hospital. Serum hs-CRP was determined using the high-sensitive Human C-reactive protein (CRP) ELISA kit catalogue number 3220: a quantitative sandwich ELISA using monoclonal antibodies against human CRP, according to the manufacturer´s protocol [27].

**Sample size justification:** the primary exposure of the study was the levels of the biomarker hs-CRP. The sample size was calculated using the formula for comparative research studies between two groups:

$$\text{N}=\frac{{\left(Z_{\text{crit}}+Z_{\text{pwr}}\right)}^{2}4\delta^{2}}{D^{2}}$$

Let N represent the total sample size, the sum of the sizes of both comparison groups, a value of δ used in the study was 2.2 mg/L; which indicates the SD of each group, assumed to be equal for both groups, the critical Z value is 1.960 as given in tables for standard normal deviation (Zcrit) corresponding to the desired significance criterion of 0.05 or 95% confidence interval (CI), the Zpwr value is 0.842 as given in standard normal deviation (Zpwr) tables corresponding to 80% statistical power, where D represents the minimum anticipated difference between two means which was estimated at 1.62 mg/L [28]. The calculated sample size (N), was 58, adjusted to 72 assuming a 20% non-response rate. The sample size of 72 at 80% power was used to detect a minimum diagnostically important difference in the levels of hs-CRP in pre-eclamptic and normotensive pregnant women of 1.62 mg/L [29], assuming a standard deviation of 2.2 mg/L, using a two-tailed t-test of the difference between means with 95% confidence intervals. Though the sample size calculated was 72, a total of 80 pregnant women were enrolled.

**Statistical analysis:** descriptive and analytical statistics were used. Bivariate analyses of the outcome (pre-eclampsia) were assessed against exposure variables (hs-CRP), HIV infection, and clinical and sociodemographic variables. After testing appropriate assumptions, bivariate analyses (Pearson Chi-square test, Fisher's exact test) were conducted to examine differences by the pre-eclampsia status for categorical variables. For the age, hs-CRP, urea, and creatinine levels, there was no evidence to suggest that the distribution was symmetric (confirmed graphically using Q-Q plots). Therefore, the results were reported as median with interquartile range (IQR) and used a nonparametric Wilcoxon Ranksum (Mann-Whitney) test to assess for any differences by pre-eclampsia status.

Univariable conditional logistic regression models were conducted to assess the association between hs-CRP and pre-eclampsia. Sociodemographics and clinical and obstetric characteristics were adjusted for in the multivariable analysis with pre-eclampsia as an outcome. First, all important predictors with a p-value less than 20% from the univariable model were added (model 1, Table 1). Then, we restricted to the HIV-negative-only group in the second step (model 2, Table 1). In step 3, we restricted to the HIV-positive-only group (model 3, Table 1). For all three models, backward stepwise regression techniques were used until the models appeared parsimonious. Interactions/effect modification was assessed between hs-CRP and significant modifying variables and did not reach any statistical significance. Multicolinearity was assessed and none of the variance inflation factors (VIFs) were above 5 suggesting that multicollinearity was not a problem. The Hosmer Lemeshow test was used to assess the model's goodness of fit. The odds ratios are presented at the significance level of 5% and 95% confidence intervals. All statistical analyses were conducted in Stata/BE, version 17 (Stata Corporation, College Station, Texas, USA).

**Table 1:** adjusted associations between predictors and pre-eclampsia

Variable		Pre-eclampsia		
		Model 1	Model 2	Model 3
	COR (95% CI)	AOR(95% CI)	AOR (95% CI)	AOR(95% CI)
Hs-CRP	1.01(1.01, 1.01)***	1.01(1.01, 1.01)***	1.01(1.01, 1.01)**	1.01(1.01, 1.01)*
Parity				
Nulliparous	Ref	Ref	Ref	Ref
Multiparous	0.58(0.21, 1.63)	0.16(0.03, 0.80)*	0.10(0.01, 1.21)	0.13(0.01, 2.38)
Marital status				
Unmarried	Ref	Ref	-	Ref
Married	0.62(0.24, 1.63)	0.15(0.03, 0.71)*		0.03(0.01, 0.33)**
HIV-serostatus				
Negative	Ref	Ref	-	-
Positive	1.01(0.42, 2.40)	0.26(0.07, 0.99)*		


Boldface indicates statistical significance (*p<0.05, **p<0.01, ***p<0.001); COR-Crude odds ratio; AOR-adjusted odds ratio; model 1-overall adjusted model unstratified; model 2-adjusted model for the HIV-negative group only; model 3-adjusted model for the HIV-positive group only. All three models were fitted with pre-eclampsia as an outcome variable, HsCRP-high sensitivity C-reactive protein as the primary exposure

**Ethical consideration:** the approval was obtained from the biomedical research ethics committee of the University of Zambia: approval number REF. 1473-2021, the National Health Research Authority (Ref No: NHRA000004/15/10/2021) and the Ministry of Health in Lusaka province (PHOLsk/101/8/1). Additionally, permission was obtained from the Senior Medical Superintendent of the Women and Newborn Hospital's office to conduct the study at the hospital. The participants were given written consent forms. Identity numbers were assigned to the datasheets and samples of the participants to protect their identities. Only willing participants were recruited, and the information obtained from the participants was confidential. All data collected was stored in locked cardboard and a password-protected private computer.

## Results

**Clinical, obstetric, and sociodemographic characteristics:** a total of 80 pregnant women with a median age of 30 years (interquartile range [IQR]=27.5, 33.5) were enrolled, of whom half, 40 (50.0%), had pre-eclampsia (20 living with HIV, 20 without HIV), and the other 40 (50.0%) were normotensive (20 living with HIV, 20 without HIV) (Table 2). A larger proportion, 56 (70.0%), were married, and 60 (75.0%) were multiparous. In addition, 39 (48.8%) were overweight, and 42(52.5%) were above 30 weeks of gestational age. The median levels for hs-CRP were 6.75mg/ml (IQR=4.99, 7.84), Urea was 2.35 mmol/l (IQR=1.86, 2.89) and creatinine was 45.0 μmol/l (IQR=41.8, 48.3).

**Table 2:** cross-tabulations: clinical and sociodemographic characteristics by pre-eclampsia status

Variable	Total population n=80, (%)	Normotensive, n=40	Pre-eclamptic, n=40	p-value
Hs-CRP (mg/ml)	6.75(4.99, 7.84)	6.13(3.96, 6.66)	7.84(7.33, 8.41)	<0.001
Age (years)	30(27.5, 33.5)	30.5(27, 35)	30(28, 33)	0.937
Urea(mmol/l)	2.35(1.86, 2.89)	1.87(1.84, 2.80)	2.47(1.87, 2.91)	0.083
Creatinine (µmol/l)	45.0(41.8, 48.3)	43(41.5, 47.1)	45.7(42.7, 49.8)	0.059
**Marital status**				
Unmarried	24(30.0)	10(25.0)	14(35.0)	0.329
Married	56(70.0)	30(75.0)	26(65.00	
**Gestational age**				
20-30	38(47.5)	18(45.0)	20(50.0)	0.654
Above 30	42(52.5)	22(55.0)	20(50.0)	
BMI kg/m^2				
Normal	29(36.3)	17(42.5)	12(30.0)	0.329
Overweight	39(48.8)	19(47.5)	20(50.0)	
Obese	12(15.0)	4(10.0)	8(20.0)	
Parity				
Multiparous	60(75.0)	28(70.0)	32(80.0)	0.302
Nulliparous	20(25.0)	12(30.0)	8(20.0)	

All values are frequencies (percentages) or median (interquartile range) unless otherwise specified, P-values from Pearson’s Chi-square test or Wilcoxon Ranksum test, BMI-body mass index, Hs-CRP-high-sensitivity reactive protein

**Figure 1:**
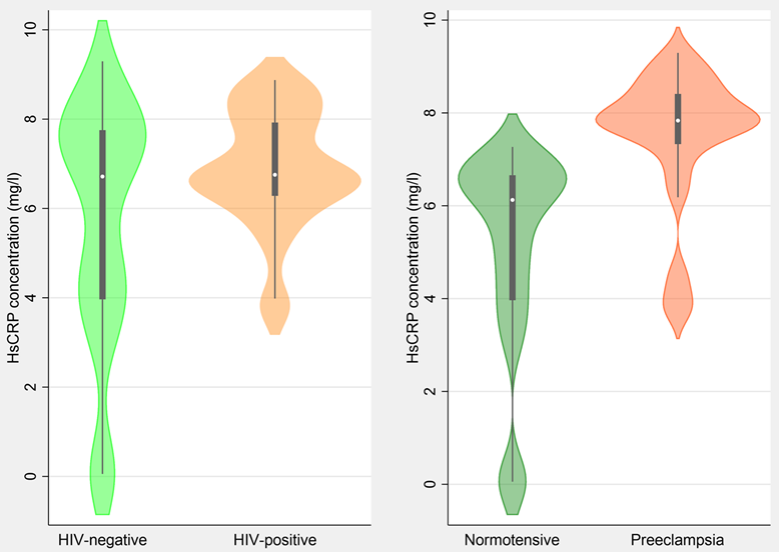
the concentration of maternal serum high sensitivity C-reactive protein (HsCRP) (mg/l) between HIV-positive and negative women and those with/without preeclampsia

**Hs-CRP levels among participants:** at bivariate analysis, pregnant women with pre-eclampsia had higher levels of hs-CRP than normotensive women (7.84mg/ml vs. 6.13mg/ml, p<0.001) (Table 2, Figure 1). In sub-group analysis, (Table 3, Figure 1), Similar hs-CRP levels were observed among pre-eclamptic women living with HIV on ART compared to HIV-negative women (7.92mg/ml vs 7.17mg/ml, p=0.862). On the other hand, normotensive women living with HIV on ART had different hs-CRP levels than HIV-negative women (6.60mg/ml vs 3.96mg/ml, p<0.001).

**Table 3:** cross-tabulation: pre-eclampsia and participant characteristics stratified by HIV-serostatus

Variable	Normotensive			Pre-eclampsia		
	HIV-negative, n=20	HIV-positive, n=20	p-value	HIV-negative, n=20	HIV-positive, n=20	p-value
Hs-CRP (mg/ml)	3.96(1.53, 5.59)	6.60(6.13, 6.75)	<0.001	7.17(7.03, 8.06)	7.92(6.93, 8.49)	0.862
Marital status						
Unmarried	8(40.0)	2(10.0)	0.028	1(5.0)	13(65.0)	<0.001
Married	12(60.0)	18(90.0)		19(95.0)	7(35.0)	
Gestational age						
20-30	9(45.0)	9(45.0)		11(55.0)	9(45.0)	0.527
Above 30	11(55.0)	11(55.0)	1.001	9(45.0)	11(55.0)	
BMI kg/m^2						
Normal	9(45.0)	8(40.0)	0.546	7(35.0)	5(25.0)	0.913
Overweight	8(40.0)	11(55.0)		9(45.0)	11(55.0)	
Obese	3(15.0)	1(5.0)		4(20.0)	4(20.0)	
Parity						
Nulliparous	7(35.0)	5(25.0)	0.366	4(20.0)	4(20.0)	1.001
Multiparous	13(65.0)	15(75.0)		16(80.0)	16(80.0)	
Age (years)	30.5(26, 33.5)	31(27, 36)	0.888	30(28, 32.5)	30.5(26.5, 33.5)	0.984
Creatinine (µmol/l)	42.2(41.5, 45.5)	45.2(42.1, 50.6)	0.115	49.0(44.3, 54.3)	43(42, 46)	0.003
Urea (mmol/l)	1.87(1.81, 2.50)	2.44(1.85, 2.80)	0.401	2.75(1.93, 3.45)	2.09(1.86, 2.85)	0.078


All values are frequencies(percentages) or median (interquartile range) unless otherwise specified, P-values from Pearson’s Chi-square test or Fisher's Exact test or Wilcoxon Ranksum test, BMI-body mass index

**Factors associated with preeclampsia:** in univariable conditional logistic regression (Table 1), a unit increase in hs-CRP was significantly associated with increased odds of pre-eclampsia. In multivariable analysis (Table 1), hs-CRP's effect on pre-eclampsia was sustained (AOR=1.01, 95% CI=1.01, 1.01) after adjusting for important predictors (model 1). On the other hand, pre-eclampsia was less common among women living with HIV infection (AOR=0.26, 95% CI=0.07, 0.99), married (AOR=0.15, 95% CI=0.03, 0.71), and multiparous (AOR=0.16, 95% CI=0.03, 0.80). In the second step (model 2), when restricted to the HIV-negative group parity attenuated the relationship between hs-CRP and pre-eclampsia, but significance was retained. In the third step (model 3), when restricted to women living with HIV infection, parity, and marital status further weakened the strength of the relationship between hs-CRP and pre-eclampsia.

## Discussion

This study aimed to investigate whether hs-CRP levels differ between women who develop pre-eclampsia compared with controls overall and in subgroups of women living with and without HIV. High-sensitivity C-reactive protein, a marker of inflammation was higher among the cases than controls and this difference was not observed in the subgroup of women living with HIV infection. Additionally, pregnant women with higher levels of hs-CRP were more likely to have pre-eclampsia. Conversely, women who were living with HIV infection, married, and multiparous were less likely to present with pre-eclampsia.

Our study's high hs-CRP levels in pre-eclamptic women are consistent with similar studies [29-32]. Two independent studies conducted by Vijayalakshmi *et al*. [31] in India and Adediji *et al*. [33] in Nigeria investigated hs-CRP levels in pregnant women with preeclampsia. In both studies, hs-CRP levels were significantly elevated in the pre-eclamptic group compared to the normotensive pregnant women. These findings provide consistent evidence that hs-CRP is associated with pre-eclampsia. The link is thought to be through the leakage of blood components such as plasma proteins and other oxidative stress products to the systemic circulation caused by endothelial damage, oxidative stress, and placental factors [34]. The placenta releases various substances into the maternal bloodstream, including placental pro-inflammatory cytokines such as IL-6 and TNF-α that stimulate the liver to release more hs-CRP, leading to increased levels of hs-CRP [31]. Therefore, routine monitoring of hs-CRP levels during pregnancy could aid healthcare providers in identifying women at higher risk of pre-eclampsia and tailoring their care accordingly.

Pathophysiological conditions such as placental hypoxia leads to oxidative stress, which increases hs-CRP production [33,35]. Oxidative stress is one of the characteristics of pre-eclampsia, which occurs when there is an imbalance between the production and elimination of the oxygen-reactive species [33] and can lead to tissue damage and inflammation which increases the levels of hs-CRP. The increased levels of hs-CRP in pre-eclamptic pregnant women inhibit nitric oxide synthetase expression. This results in decreased nitric oxide (NO) production, leading to endothelial dysfunction and impaired blood flow [29,36]. High-sensitivity C-reactive protein inhibit eNOS by inhibiting GTP cyclohydrolase 1 through the p38 kinase pathway, this enzyme is important in the first step in the de novo synthesis of tetrahydrobioterin, an important cofactor for eNOS [37].

The inhibition of eNOS by hs-CRP may contribute to higher vascular resistance and, therefore be involved in preeclampsia [38]. Studies by Adediji *et al*. [33] and Vijayalakshmi *et al*. [31] support the proposition that a reduction in NO production hinders endothelium-dependent vasorelaxation, disrupting vascular homeostasis and triggering endothelial dysfunction. This dysfunction is a characteristic feature in many cardiovascular diseases, including hypertension and atherosclerosis [39], and is a key feature of preeclampsia in pregnant women [40].

In recent investigations, HIV infection and the use of ART have been implicated in the increased risk of developing pre-eclampsia [13,41]. In the present study, pregnant women living with HIV infection on ART were less likely to develop pre-eclampsia. These findings suggest that effective ART treatment could reduce the viral load in the body [42], which helps to mitigate chronic inflammation, improves endothelial dysfunction [43], and restore immune function which contributes to a more balanced immune response during pregnancy [17], thereby, reducing the risk of pre-eclampsia. The precise mechanism underlying the protective effect of HIV infection and ART remains unclear. A study conducted by Mukosha *et al*. [3] in Zambia, corroborates our findings, demonstrating an elevated prevalence of pre-eclampsia among HIV-uninfected pregnant women at 8.4%, in contrast to HIV-infected pregnant women on ART at 4.8%. The observed protective effect in HIV-infected pregnant women on ART against pre-eclampsia warrants further investigation into potential underlying mechanisms, which may hold promise for developing novel preventive strategies.

The apparent protective effect of HIV infection on pre-eclampsia occurrence was observed when the analysis was restricted to the women living with HIV where the levels of hs-CRP were not significantly different between pre-eclamptic women and normotensive counterparts. However, regression analysis showed that hs-CRP remains a relevant biomarker for pre-eclampsia risk prediction irrespective of HIV serostatus, and its predictive value should not be overlooked in HIV-positive pregnant women.

Additionally, it was observed that pregnant women who had experienced multiple pregnancies were less prone to developing pre-eclampsia. The study's results align with the findings of Maeda *et al*. [44], who similarly noted a lower incidence of pre-eclampsia among multiparous pregnant women. One possible explanation for this phenomenon is immunological reasons [4,5], through each pregnancy, a woman's body encounters foreign antigens from the father, fostering the development of immunological tolerance.

In the present study, unmarried pregnant women were more likely to develop pre-eclampsia than those who were married. We speculate that a plausible explanation may not typically be a direct biological factor but is likely to be associated with lifestyle and psychosocial factors such as chronic stress [46]. Healthier lifestyles and social support can potentially improve the psychological well-being of pregnant women by enhancing their stress-coping ability and alleviating stressful conditions, leading to lower levels of systemic inflammation and hs-CRP, as observed in married pregnant women [46,47]. The study by Phiri and Nyamaruze [48], found that social support helps with stress during pregnancy and is important throughout the different phases of the pregnancy. Integrating these predictors alongside hs-CRP and HIV status can lead to a more comprehensive risk assessment for pre-eclampsia in pregnant women.

**Strengths and limitations:** to the best of our knowledge, this was the first report of hs-CRP levels in pregnant women who develop pre-eclampsia in Zambia. The results offer baseline information on the levels of hs-CRP in this population and could be useful for larger mechanistic studies relating to hypertensive disorders of pregnancy. The study also highlights important findings, such as the increased likelihood of pre-eclampsia in pregnant women with higher levels of hs-CRP and the decreased likelihood of pre-eclampsia in married and multiparous pregnant women. However, these findings should be interpreted in the context of some limitations. Firstly, as it was a hospital-based study, the findings may not be generalizable to the entire province or country. Secondly, the study could not assess the independent effect of ART on pre-eclampsia due to the current guidelines on HIV management in this setting.

## Conclusion

High-sensitivity C-reactive protein levels were higher among the cases than controls. The difference was not observed in the subgroup of women living with HIV on ART. Participants with high hs-CRP levels had the highest odds of preeclampsia, suggesting that hs-CRP may be useful in predicting preeclampsia. The ethnogeographic variations in the relationship between hs-CRP and pre-eclampsia merit further investigations. Furthermore, the discovery of assays that can accurately identify those pregnant women at the highest risk for pre-eclampsia early in gestation could improve maternal and neonatal outcomes.

### What is known about this topic

- *Pre-eclampsia and HIV infection are significant health problems worldwide;*
- *There have been conflicting results regarding the risk of developing pre-eclampsia in high HIV settings;*
- *Pre-eclampsia is characterized by inflammation.*

### What this study adds

- *Women living with HIV infection are less likely to present with pre-eclampsia;*
- *High-sensitivity C-reactive protein levels in women with pre-eclampsia in a setting with a high HIV burden;*
- *Pregnant women with high levels of hs-CRP have higher odds of developing pre-eclampsia.*
